# Mutation allele frequency threshold does not affect prognostic analysis using next-generation sequencing in oral squamous cell carcinoma

**DOI:** 10.1186/s12885-018-4481-8

**Published:** 2018-07-24

**Authors:** Jie Ma, Yong Fu, Yao-yao Tu, Ying Liu, Yi-ran Tan, Wu-tong Ju, Curtis R. Pickering, Jeffrey N. Myers, Zhi-yuan Zhang, Lai-ping Zhong

**Affiliations:** 1 Department of Oral & Maxillofacial-Head & Neck Oncology, Ninth People’s Hospital, Shanghai Jiao Tong University School of Medicine, No 639, Zhizaoju Rd, Shanghai, 200011 China; 2 Department of Head & Neck Surgery, University of Texas MD Anderson Cancer Center, Houston, USA

**Keywords:** Oral squamous cell carcinoma, Next-generation sequencing, Mutation allele frequency

## Abstract

**Background:**

With the development of sequencing technologies, there may be some disputes on sequencing analysis. The aim of this study was to investigate different allele frequency thresholds of mutations in targeted genes on prognostic analyses using a panel of cancer associated gene exons (CAGE) in oral squamous cell carcinoma (OSCC).

**Methods:**

Forty-six patients were included in this study. Twelve genes were sequenced and analyzed using next-generation sequencing from formalin-fixed paraffin-embedded tissues. Allele frequency thresholds of 10, 5, and 3% were used for prognostic analyses.

**Results:**

With a mean sequence depth of 3199-fold, 99% of CAGE were represented by at least 10 reads. Ninety-four non-synonymous (missense [70.2%], nonsense [11.7%], splice site [10.6%], and insertion/deletion [7.5%]) mutations were detected in 40 OSCC patients with an allele frequency threshold of 10%. *TP53* (78.3%), *NOTCH1* (30.4%), *CASP8* (13.0%), *CDKN2A* (10.9%), and *CDH1* (6.5%) were the most frequently mutated genes. Using allele frequency thresholds of 10, 5, and 3%, there were no significant differences in clinical outcomes between patients with non-synonymous mutations and wild type genotypes.

**Conclusions:**

*TP53*, *NOTCH1*, *CASP8*, *CDKN2A*, and *CDH1* are the most frequently mutated genes in OSCC patients. The allele frequency threshold used in this study does not affect the results of clinical outcome analysis.

**Electronic supplementary material:**

The online version of this article (10.1186/s12885-018-4481-8) contains supplementary material, which is available to authorized users.

## Background

Oral squamous cell carcinoma (OSCC) comprises a major subset of head and neck cancer and has a 5-year survival rate of approximately 50 to 60%, with lower survival rates for patients who present at late clinical stages [1]. For decades, the standard treatment for patients with locally advanced OSCC has been surgery followed by post-operative radiotherapy or concurrent chemo-radiotherapy [2]. Although efforts have been made to improve treatment modalities and decrease treatment-related morbidity, patient outcomes have not improved. Therefore, it is essential to understand different aspects of the molecular characteristics underlying this disease to better understand its behavior and identify more effective ways to treat it.

Next-generation sequencing (NGS) includes sequencing of the whole exome or targeted genes, enabling the identification of causative mutations, which are targets for therapy [3–7]. Previously, genomic analyses using NGS have detected mutations in several genes critical for tumor growth and survival as well as identified targeted hotspots of other genes. Unfortunately, some of these studies did not report matched normal control sample sequences in addition to those of the tumor specimens, which can make it challenging to identify germline mutations that could contribute to the development of these cancers [5, 6, 8, 9].

Using state-of-the-art sequencing techniques, sequence coverage can reach as high as 3000-fold or more for most regions of the genome. However, several potential bottlenecks remain for accurate sequencing, such as sequence coverage and the allele frequency of targeted gene mutations. For NGS studies of cancer samples, the allele frequency represents the percentage of sequence reads carrying a mutant allele of an individual patient’s cancer, which can be influenced by many factors. These include the infiltration or contamination of tumors with lymphocytes, stromal tissues, and/or necrotic/infected tissue, and tumor heterogeneity. Therefore, investigators have arbitrarily set thresholds for defining the variant allele frequency as mutation. For allele frequency, no universal standard has been established for the allele frequency threshold for sequencing analysis, and thresholds of 10, 5, and 3% have been reported in the literature [5, 10, 11]. Furthermore, whether different allele frequencies have an impact on the prognosis of cancer patients has yet to be reported.

In this study, a panel of cancer associated gene exons (CAGE) including 12 genes (*TP53*, *NOTCH1*, *CASP8*, *CDKN2A*, *CDH1*, *ANXA1*, *EGFR*, *IGFBP3*, *TGFB1*, *CTNNB1*, *PTEN*, and *TP63*) were analyzed using NGS in 46 OSCC patients with both cancerous tissues and matched non-cancerous tissues. Prognostic analysis was performed after calling non-synonymous mutations using allele frequency thresholds of 10, 5, and 3%.

## Methods

### Patients and samples

Forty-six patients with locally advanced OSCC from 2008 to 2010 were included in this study, which was approved by the Human Research Ethics Committee of the Ninth People’s Hospital Shanghai Jiao Tong University School of Medicine [approval number: 2008(12)]. Cancerous tissue samples were derived from biopsy specimens, while matched non-cancerous tissue samples were derived from neck dissection specimens; all specimens were formalin-fixed and paraffin-embedded (FFPE). All patients received radical surgery and post-operative radiotherapy. Four of the patients received docetaxel, cisplatin, and 5-fluorouracil (TPF) induction chemotherapy prior to definitive treatment with surgery and radiotherapy.

### DNA extraction and quantification

All tissue specimens were reviewed by two pathologists, and tumor cell areas on hematoxylin-eosin (HE) stained slides were determined for manual microdissection and subsequent DNA sequencing. Only tumor cells (appearing as HE-stained) were microdissected as cancerous samples, with an average tumor purity of 85% (ranging from 80 to 95%). Five 10-μm FFPE sections from each block were deparaffinized, and DNA was extracted using the QIAamp DNA FFPE Tissue Kit (Qiagen, Hilden, Germany). Quality and quantity of the purified DNA were measured using the Qubit and Nano-Drop platforms (Thermo Fisher Scientific, Waltham, MA, USA).

### Deep sequencing of PCR amplicons

Ten nanograms of DNA was used for multiplex PCR amplification. Libraries were constructed using the Ion AmpliSeq Library Kit v2.0 (Thermo Fisher Scientific) according to the manufacturer’s instructions. The quality of the obtained libraries was evaluated using Agilent 2100 Bioanalyzer on-chip electrophoresis (Agilent Technologies, Palo Alto, CA, USA). Emulsion PCR was performed with the OneTouch DL or OneTouch 2 system (Thermo Fisher Scientific). Sequencing was run on the Ion Torrent Personal Genome Machine (Thermo Fisher Scientific), loaded with a 316 or 318v2 chip as per the manufacturer’s protocol. Data analysis, including alignment to the hg19 human reference genome as well as variant calling and filtering, was completed using Torrent Suite Software v3.6 (Thermo Fisher Scientific). Filtered variants were annotated using Ion Reporter software v4.4 (Thermo Fisher Scientific).

### Mutations confirmation

Selected mutations were confirmed using Sanger sequencing. Sequence variants were compared with the head and neck squamous cell carcinoma dataset from the Cancer Genome Atlas (TCGA), dbSNP1000 Genomes, ClinVar database, COSMIC, 5000 Exomes, OMIM, and Pfam databases. SIFT, Polyphen, Phylop, and Grantham scores were used to estimate evolutionary conservation and the effects of amino acid substitutions on the structure and function of the protein.

In the context of this analysis, a somatic mutation was considered to be “validated” if: (1) the altered reads were ≥ 10, (2) non-synonymous mutations had an allele frequency ≥ 3%, and (3) non-synonymous mutations were not found in any of the SNP databases. All mutations were visually confirmed using the Integrative Genomics Viewer (IGV v2.3).

### Statistical analysis

Overall survival (OS) was calculated from the date of the pathological diagnosis to the date of death; disease-free survival (DFS), locoregional recurrence-free survival (LRFS), and distant metastasis-free survival (DMFS) were calculated from the date of the pathological diagnosis to recurrence, locoregional recurrence, or distant metastasis or death from any cause, respectively. For descriptive analyses, categorical data were expressed as number and percentage. Survival analysis was conducted using the Kaplan-Meier method with a Log-rank test. Hazard ratios (HRs) were calculated using the Cox proportional hazards model. All hypothesis-generating tests were two-sided at a significance level of 0.05. Data were analyzed with SPSS v. 18.0 for Windows (SPSS Inc., Chicago, IL, USA).

## Results

### Sequencing results

Among the targeted CAGE (for *TP53*, *NOTCH1*, *CASP8*, *CDKN2A*, *CDH1*, *ANXA1*, *EGFR*, *IGFBP3*, *TGFB1*, *CTNNB1*, *PTEN*, and *TP63*), a mean of 3099-fold sequence coverage was achieved, and 99% of the targeted CAGE was represented by at least 10 reads. Compared to matched non-cancerous tissues and reference sequences, a total of 94 non-synonymous (missense, nonsense, splicing site, insertion, and deletion) mutations were detected in the cancerous tissue samples from 40 OSCC patients with an allele frequency threshold of ≥10%. None of the non-synonymous mutations were found in the SNP databases. The average number of non-synonymous mutations per sample was 2.04. Missense mutations made up the majority (70.2%) of the identified variants, followed by nonsense mutations (11.7%), insertions/deletions (10.6%), and splice site mutations (7.5%) (Additional file 1: Table S1).

With an allele frequency threshold of ≥5%, 132 non-synonymous mutations were detected in the cancerous tissue samples from 41 patients. The average number of non-synonymous mutations per sample was 2.87. Missense mutations made up the majority (77.3%) of the identified variants, followed by nonsense mutations (9.9%), insertions/deletions (7.6%), and splice site mutations (5.3%) (Additional file 2: Table S2). With an allele frequency threshold of ≥3%, 239 non-synonymous mutations were detected in the cancerous tissue samples from 42 patients. The average number of non-synonymous mutations per sample was 5.20. Similar to the 10 and 5% thresholds, missense mutations made up the majority (86.2%) of the identified variants followed by nonsense mutations (6.7%), insertions/deletions (4.2%), and splice site mutations (2.9%) (Additional file 3: Table S3).

To verify the reliability of the deep sequencing in our panel, the remaining genomic DNA was used for validation. *TP53* mutation was selected as a representative example because it was the most frequently mutated gene with 46 genetic variates in our panel. The *TP53* Sanger sequencing was performed to confirm the *TP53* variants. Because of the low DNA content, 9 non-synonymous mutations cannot be validated by Sanger sequencing; among the other 37 non-synonymous mutations, 70.3% (26/37) of them were successfully validated by Sanger sequencing in the same DNA samples (Additional file 4: Table S4).

### Mutation landscape in the targeted genes

Non-synonymous mutations were identified in all 12 genes when the allele frequency threshold was not defined. However, when the allele frequency threshold was defined as ≥10%, the most frequently mutated genes were *TP53*(78.3%), *NOTCH1*(30.4%), *CASP8*(13.0%), *CDKN2A*(10.9%), and *CDH1*(6.5%). Genetic mutations in the most frequently mutated genes were identified in 40 (87.0%) of the patients. The mutation frequency was very low in *ANXA1* (2.2%), *EGFR* (2.2%), *IGFBP3* (2.2%), and *TGFB1* (2.2%). No non-synonymous mutations were observed at an allele frequency of ≥10% in *CTNNB1*, *PTEN*, and *TP63*. The mutation frequency of each gene when the allele frequency threshold was defined as ≥5% and ≥ 3% is shown in Table 1.

**Table 1:** Mutant frequency of targeted genes in the 46 patients with oral squamous cell carcinoma

Gene	AF^a^ ≥ 3%	AF ≥5%	AF ≥10%	TCGA HNSCC database	*p*-value^b^ (AF ≥ 10%)
*ANXA1*	1/46 (2.2%)	1/46 (2.2%)	1/46 (2.2%)	1/279 (0.4%)	0.263
*CASP8*	9/46 (19.6%)	7/46 (15.2%)	6/46 (13.0%)	23/279 (8.2%)	0.272
*CDH1*	6/46 (13.0%)	4/46 (8.7%)	3/46 (6.5%)	4/279 (1.4%)	0.062
*CDKN2A*	7/46 (15.2%)	5/46 (10.9%)	5/46 (10.9%)	60/279 (21.5%)	0.095
*CTNNB1*	3/46 (6.5%)	3/46 (6.5%)	0/46 (0)	2/279 (0.7%)	1.000
*EGFR*	2/46 (4.3%)	2/46 (4.3%)	1/46 (2.2%)	9/279 (3.2%)	1.000
*IGFBP3*	3/46 (6.5%)	2/46 (4.3%)	1/46 (2.2%)	1/279 (0.4%)	0.263
*PTEN*	1/46 (2.2%)	0/46 (0)	0/46 (0)	5/279 (1.8%)	1.000
*TGFB1*	4/46 (8.7%)	2/46 (4.3%)	1/46 (2.2%)	1/279 (0.4%)	0.263
*TP63*	4/46 (8.7%)	1/46 (2.2%)	0/46 (0)	5/279 (1.8%)	1.000
*TP53*	36/46 (78.3%)	36/46(78.3%)	36/46 (78.3%)	202/279 (72.4%)	0.406
*NOTCH1*	23/46 (50.0%)	16/46(34.8%)	14/46 (30.4%)	51/279 (18.3%)	0.056

^a^AF: allele frequency

^b^The difference between the mutation rates observed in our cohort and those in the TCGA HNSCC database

Note: Mutation frequency provided how often a mutation may be expressed in a particular genetic population. Allele frequency is the relative frequency of an allele of a gene at a particular locus in a population. Non-synonymous mutations were identified in all the 12 genes if the threshold of allele frequency was defined as ≥3%, ≥5% and ≥ 10%. When compared to the mutational patterns reported in The Cancer Genome Atlas (TCGA) head and neck squamous cell carcinoma (HNSCC) database (containing the whole-exome sequencing data from 279 samples), with the threshold of allele frequency of ≥10%, the frequency of mutations detected in our study was similar to TCGA database, with the exception of *NOTCH1*and *CDH1*

We then compared our data to the mutational patterns reported in The Cancer Genome Atlas (TCGA) head and neck squamous cell carcinoma (HNSCC) database (containing whole-exome sequencing data from 279 samples) (Table 1). With an allele frequency threshold of ≥10%, the frequency of mutations detected in our study was similar to that reported in the TCGA database, with the exception of *NOTCH1* and *CDH1.* The mutation frequencies of *NOTCH1* and *CDH1* were much higher in our cohort than those reported in the TCGA database (30.4% vs. 18.3%, *P* = 0.056 and 6.5% vs. 1.4%, *P* = 0.062 for *NOTCH1* and *CDH1*, respectively). Further analysis on the molecular characteristics of the detected mutations was based on an allele frequency threshold of ≥10%.

### Molecular characteristics of the detected mutations

*TP53* was the most frequently mutated gene, with a total of 46 genetic variants (33 missense mutations, 4 nonsense mutations, 5 insertions/deletions, and 4 splice-site mutations) in 36 patients (78.3%). Thirty-three out of 46 (71.7%) p53 mutations were found to be located in the DNA-binding domain, while 4 (8.7%) mutations were in the tetramerization motif, and 2 mutations (4.4%) were in the transactivation motif (Table 2). When compared to the TCGA database, p.Val216Met, p.Pro151Thr, p.Arg175His, p.Arg337Cys, p.Arg282Trp, p.Ala159Val, p.Arg273His, p.Arg248Gln, p.Arg282Trp, p.His193Leu, p.His178fs, p.Gly245Ser, p.Pro152Leu, p.Tyr220Cys, p.Gln331Ter, p.Pro151His, p.Arg342Ter, p.Glu286Lys, and p.Arg213Ter appeared in the TCGA HNSCC database; p.Cys135Phe, p.Phe113Cys, p.Cys176Phe, p.Trp53Ter, p.His179Leu, p.Val272Leu, p.Cys135Tyr, p.Arg213Gln, p.Pro191del, p.Val274Phe, p.Thr253Ile, p.Asp184His, p.Cys135Phe, p.Val218Glu, p.Ile255Phe, p.Asp148Asn, p.Asp57Asn, p.Pro128Ser, p.Leu93fs, and p.Pro85Ser appeared in the TCGA database of breast, bladder, renal, lung, stomach cancers and melanomas. The other mutations listed in the Additional file 1: Table S1, Additional file 2: Table S2, Additional file 3: Table S3 did not appear in the TCGA database.

**Table 2:**
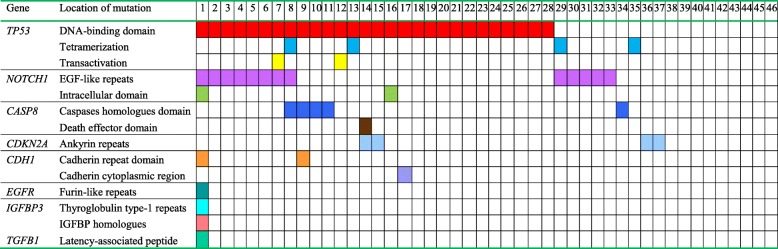
Location of non-synonymous mutations in the conserved domains in the 46 patients with oral squamous cell carcinoma

Note: Molecular characteristics of the detected mutations for the targeted genes. The p53 DNA-binding domain was the major conserved domain in 28 patients (60.9%), and the notch1 EGF-like repeats domain was the second major conserved domain in 13 patients (28.3%), followed by the caspase homology domain of caspase 8 in five patients (10.9%), tetramerization motif of p53 in four patients (8.7%), and ankyrin repeats of p16 in four patients (8.7%)

*NOTCH1* was also frequently mutated, with a total of 25 genetic variants (22 missense mutations, 2 insertions and deletions, and 1 splice site mutation) in 14 patients (30.4%). Nineteen out of 25 (76.0%) mutations were found to be located in the EGF-like repeat domains, and 5 (20.0%) mutations were in the intracellular domain, including 1 mutation in the ankyrin repeat domain (Table 2). When compared to the TCGA database, p.Ala465Thr and p.Asp338Asn appeared in the TCGA HNSCC database; p.Asn718Ser, p.Glu488Lys, p.Pro2332Leu, p.Arg2272Cys, p.Arg365Cys, p.Val1229Ile, p.Gly1195Arg, p.Pro1097Ser, p.Ala1338Thr, p.Ser2336Asn, p.Ser836Asn, p.Pro668Ser, p.Phe436Leu, p.Thr767Ile, p.Met2362Ile, p.Ser1541Asn, p.Pro148Leu, p.Arg1758Cys, p.Arg1211Gln, and p.Thr588Ile appeared in the TCGA breast, esophageal, colon, bladder, prostate, lung cancers and melanomas.

Eight *CASP8* mutations were identified in six patients (13.0%). Among them, 6 mutations were inactivating mutations (4 nonsense mutations and 2 deletions); the other 2 mutations were missense mutations. Seven (87.5%) mutations were located in the caspase homology domain, and the other was in the death effector domain (Table 2). When compared to the TCGA database, p.Arg494Ter and p.Arg472Ter appeared in the TCGA HNSCC database; p.Lys532fs and p.Gln417Ter appeared in the TCGA colorectal and bladder cancer database, respectively.

Five *CDKN2A* mutations were identified in five patients (10.4%). Most of them were inactivating mutations, including 2 nonsense mutations, 1 insertion, and 1 splice site mutation; the other was missense mutation. Four *CDKN2A* mutations were located in ankyrin repeats, and the other was a splice site mutation. When compared to the TCGA database, p.Glu120Ter appeared in the TCGA HNSCC database.

Three *CDH1* mutations were identified in three patients (6.5%). Two mutations were missense mutations and the other was a nonsense mutation. Two *CDH1* mutations were located in the cadherin repeat domain, and the other was in the cadherin cytoplasmic region. p.Trp532Ter and p.Arg90Trp appeared in the TCGA cervical and mixed cancer database, respectively.

Three *EGFR* mutations were identified in one patient (2.2%), including 2 missense mutations and 1 splice site mutation. The 2 missense mutations were located in the furin-like repeats of EGFR. Two missense *IGFBP3* mutations were identified in one patient (2.2%), and these mutations were located in the thyroglobulin type-1 repeat domain and the IGFBP homology domain, respectively. One missense *ANXA1* mutation was identified in one patient (2.2%), but it was not located in the conservative region of annexin a1. One missense *TGFB1* mutation was identified in one patient (2.2%), which was in the latency-associated peptide of TGFβ-1. Some of the mutations also appeared in the TCGA database.

### Mutations in conserved domains

The p53 DNA-binding domain was the major conserved domain that contained a mutation in 28 patients (60.9%), and the *NOTCH1* EGF-like repeats domain was the second major conserved domain, with a mutation in 13 patients (28.3%), followed by the caspase homology domain of caspase 8 that was mutated in five patients (10.9%), the tetramerization motif of p53 with a mutation in four patients (8.7%), and the ankyrin repeat domains of p16, which contained mutations in four patients (8.7%) (Table 2).

There was one patient (2.2%) who had 8 mutations in conserved domains of various genes, and one patient (2.2%) had 4 mutations in these conserved domains, followed by three patients (6.5%) with 3 mutations, 13 patients (28.3%) with 2 mutations, and 19 patients (41.3%) with just 1 mutation in a conserved domain.

### Relationships between mutations and patient characteristics as well as survival

**Fig. 1:**
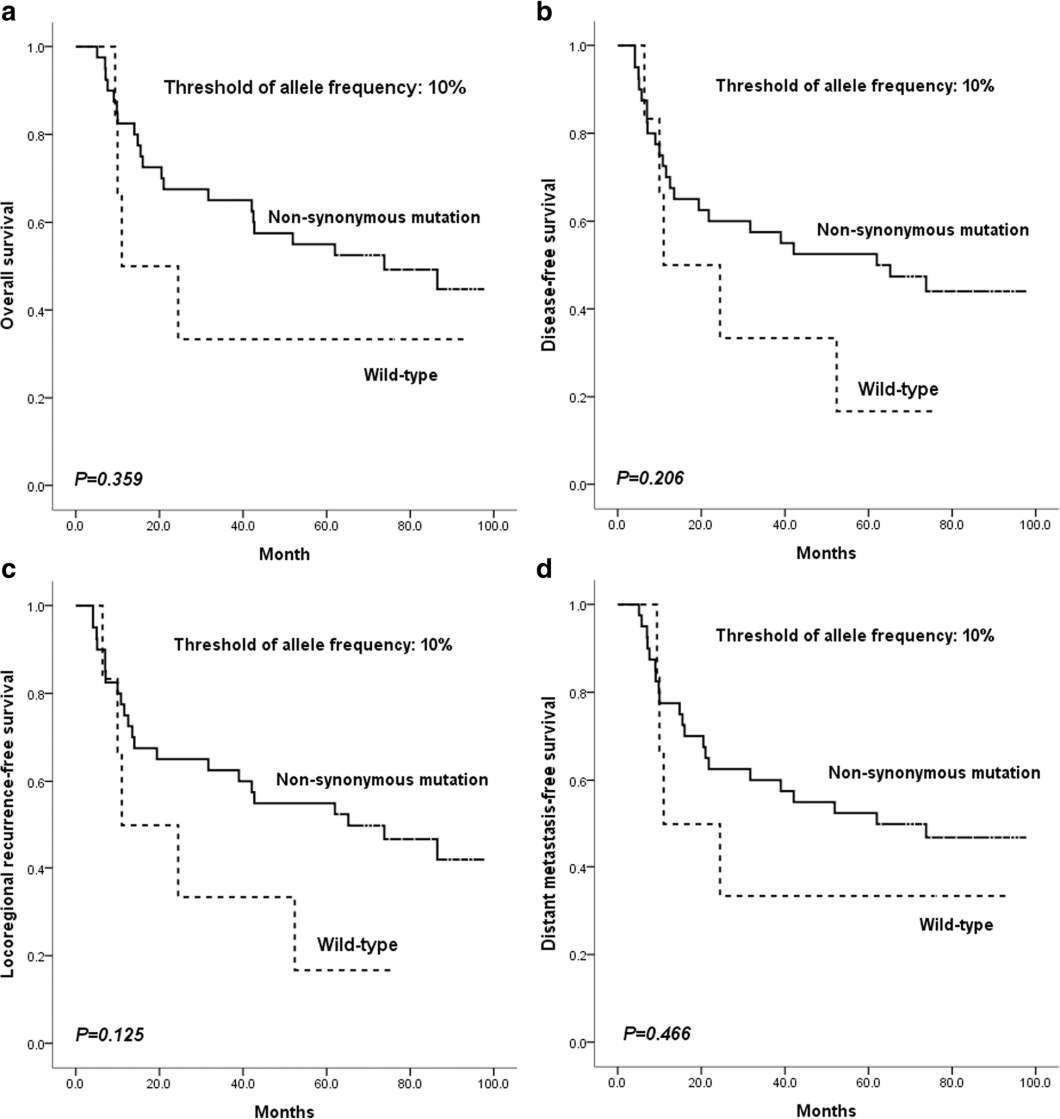
Survival comparison between patients with non-synonymous mutations (including all targeted genes of CAGE) and wild type carriers using an allele frequency threshold of 10%. The difference was not significant for overall survival (**a**), disease-free survival (**b**), locoregional recurrence-free survival (**c**), or distant metastasis-free survival (**d**)

Correlation analysis between non-synonymous mutant status (including all targeted genes of CAGE) and baseline characteristics was performed; no significant correlations were found (Table 3). Survival analysis found no significant differences in outcomes with regard to OS, DFS, LRFS, and DMFS between patients with non-synonymous mutations and wild type carriers (Fig. 1).

**Table 3:** Correlation analysis between non-synonymous mutations of all targeted genes of CAGE and baseline characteristics in the 46 patients with oral squamous cell carcinoma

Characteristics	Total patients *N* = 46	Non-synonymous mutations		*P* value*
		+	–	
	n (%)	n (%)	n (%)	
Gender				
Male	12 (26.1)	10 (25.0)	2 (33.3)	0.644
Female	34 (73.9)	30 (75.0)	4 (66.7)	
Age (years)				
< 60	27 (58.7)	23 (57.5)	4 (66.7)	1.000
≥ 60	19 (41.3)	17 (42.5)	2 (33.3)	
Site				
Tongue	19(41.3)	18(45.0)	1(16.7)	0.182
Buccal	6(13.0)	6(15.0)	0(0.0)	
Gingiva	6(13.0)	5(12.5)	1(16.7)	
Floor of mouth	3(6.5)	2(5.0)	1(16.7)	
Palate	9(19.6)	6 (15.0)	3 (50.0)	
Retromolar trigone	3(6.5)	3(7.5)	0(0.0)	
Clinical T stage				
T1/T2	13(28.3)	11(27.5)	2(33.3)	1.000
T3/T4	33 (71.7)	29(72.5)	4(66.7)	
Clinical N stage				
N0	14(30.4)	11(27.5)	3 (50.0)	0.633
N1	12(26.1)	11(27.5)	1(16.7)	
N2	20(43.5)	18(45.0)	2(33.3)	
Clinical stage				
III	22(47.8)	19(47.5)	3 (50.0)	1.000
IVA	24(52.2)	21(52.5)	3 (50.0)	
Pathological differentiation grade				
Well	13(28.3)	11(27.5)	2(33.3)	1.000
Moderately/Poorly	33(71.7)	29(72.5)	4(66.7)	
Smoking status^a^				
Current/former	19(41.3)	17(42.5)	2(33.3)	1.000
Never	27(58.7)	23 (57.5)	4(66.7)	
Alcohol use^b^				
Positive	24(52.2)	22(55.0)	2(33.3)	0.405
Negative	22(47.8)	18(45.0)	4(66.7)	

*P value from the chi-square test was reported to compare the difference between the patients with and without non-synonymous mutation of targeted genes based on different baseline characteristics

^a^Former/current smokers defined as at least a one pack-year history of smoking

^b^Positive alcohol use was defined as current alcohol use of more than one drink per day for 1 year (12 oz of beer with 5% alcohol, or 5 oz of wine with 12–15% alcohol, or one ounce of liquor with 45–60% alcohol). All other patients were classified as negative alcohol use

Note: No significant correlation was found between the non-synonymous mutant status of all targeted genes of CAGE and baseline characteristics in the 46 patients with oral squamous cell carcinoma

When the allele frequency thresholds of 5 and 3% were used, the difference in outcomes between patients with non-synonymous mutations and wild type carriers was non-significant (Additional file 5: Figure S1 and Additional file 6: Figure S2).

**Fig. 2:**
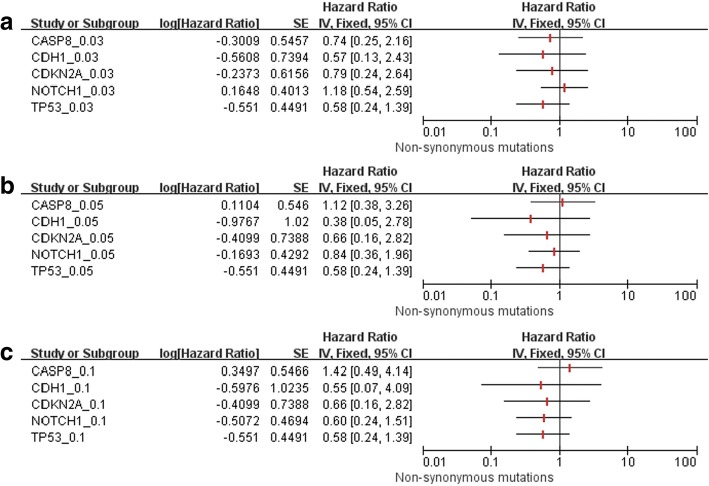
Using the allele frequencies of 3% (**a**), 5% (**b**), and 10% (**c**), there were no significant differences in overall survival between patients with non-synonymous mutations in five single genes (*TP53*, *NOTCH1*, *CASP8*, *CDKN2A,* and *CDH1*) and wild type carriers

**Fig. 3:**
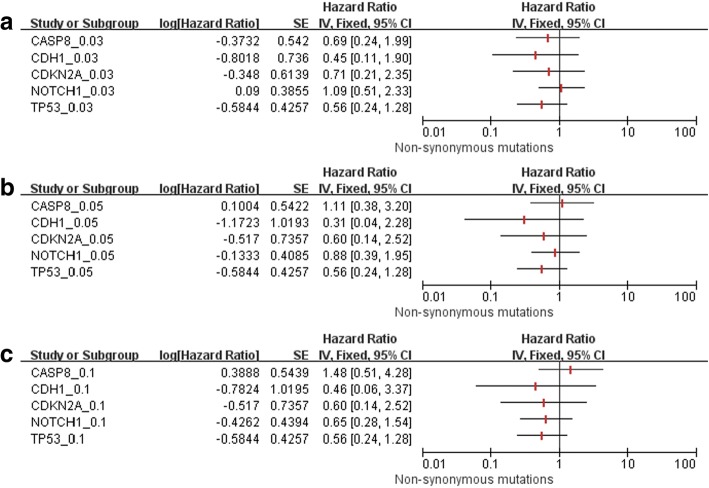
Using the allele frequencies of 3% (**a**), 5% (**b**), and 10% (**c**), there were no significant differences in disease-free survival between patients with non-synonymous mutations in five single genes (*TP53*, *NOTCH1*, *CASP8*, *CDKN2A,* and *CDH1*) and wild type carriers

Five genes (*TP53*, *NOTCH1*, *CASP8*, *CDKN2A,* and *CDH1*) with an allele frequency of non-synonymous mutations above 10% were selected for further relationship analysis, and no significant correlations were found between patients with non-synonymous mutations in each gene and baseline characteristics, with the exception of *NOTCH1* (Additional file 7: Table S5, Additional file 8: Table S6, Additional file 9: Table S7, Additional file 10: Table S8, Additional file 11: Table S9). The *NOTCH1* non-synonymous mutation rate was higher in T1/T2 patients than that in T3/T4 patients. To investigate the impact of allele frequency threshold on survival analysis, a univariate Cox model was used with allele frequency thresholds of 10, 5, and 3%; the difference in OS **(**Fig. 2**)** and DFS (Fig. 3) between patients with non-synonymous mutations and wild type carriers was not significant.

## Discussion

In the present study, we found that when using the different allele frequency thresholds of 10, 5, and 3%, the total non-synonymous mutation rate in the cancerous tissue samples was 87.0% (40/46), 89.1% (41/46), and 91.3% (42/46), respectively. The most frequently mutated genes were *TP53*, *NOTCH1*, *CASP8*, *CDKN2A*, and *CDH1*. Our analysis of the distribution of mutations in these genes will be helpful for future functional studies. We also found that using different allele frequency thresholds did not affect the prognosis of patients with and without non-synonymous mutations detected by deep NGS.

Deep NGS has been shown to detect low-frequency mutations located in known causative genes [3, 12, 13]. Several studies have found pathogenic mutations in OSCC using NGS; however, some have only targeted a few genes or hotspots, while others lacked matched normal control tissues, making it impossible to rule out germline mutations in the tumor specimens [5, 6, 8, 9]. In the present study, the mean sequence coverage of targeted CAGE reached 3099-fold, and the frequency of mutations was quite similar to that reported in the TCGA HNSCC database, with the only exception being the *NOTCH1* gene, which had a significantly higher mutation frequency in our cohort than that in the TCGA HNSCC database. The higher mutation frequency of *NOTCH1* gene has also been reported in studies in the Chinese population [8, 9]. Nevertheless, the overall frequency of targeted CAGE in this study was consistent with the TCGA HNSCC database. Some subtle differences in specific alleles may reflect a difference in ethnicity, etiology, or disease stage.

Although many efforts have been made to improve the depth of NGS coverage, there are some disputes on clinical applications, such as the sequence coverage and allele frequencies of targeted gene mutations for prognostic analysis. For allele frequency, no universal threshold has been established for sequencing analysis, and thresholds of 10, 5, and 3% have been reported in the literature [5, 10, 11]. In our study, 10, 5, and 3% allele frequencies were used as threshold for prognostic analysis, but we found that survival rates between patients with and without non-synonymous mutations were similar, not only using the panel of CAGE, but also using single CAGE genes. Therefore, there is no evidence that an allele frequency threshold affects prognosis. However, our study included a limited sample size, and therefore further investigation with larger sample sizes is recommended for determining a reasonable allele frequency threshold for clinical outcome analysis in cancer patients.

According to previous studies [3, 12], *TP53* was the most frequently mutated gene in OSCC, and 71.7% of mutations were located in the DNA-binding domain, 8.7% in the tetramerization motif, and 4.4% in the transactivation motif. The wild type p53 protein can activate gene transcription by binding to specific DNA sequences, subsequently controlling the cell cycle checkpoint, which is responsible for maintaining genomic integrity. When *TP53* mutations occur in the DNA-binding domain, the p53 protein cannot bind to these specific DNA sequences, and the rate of gene transcription changes to some extent [14–16]. Meanwhile, mutations in the tetramerization motif might deregulate the oligomerization process of p53 [15].

For *NOTCH1*, in this study, more than two-thirds of the mutations were located within EGF-like repeats, which could significantly impact NOTCH1 protein activity. EGF-like repeats contain the “ligand-binding” domain, which is the key domain for direct interactions between NOTCH1 and its ligands (Jagged1 and 2 and Delta-like 1, 3, and 4) [17, 18]. Mutations in this region can disturb signal transduction pathways since NOTCH1 signaling depends on these direct interactions. At the same time, mutations in EGF-like repeat regions can intensify NOTCH1 signaling because the integrity of EGF-like repeats is necessary to suppress this activity [19, 20]. Furthermore, the mutant NOTCH1 protein may not only lose its function, but could potentially gain new abilities like mutant p53 [21]. Further investigation is necessary with larger sample sizes to identify potential novel mechanisms of *NOTCH1* mutations in OSCC.

The majority of *CASP8* mutations were found in the caspase homology domain in the present study. Activated caspase-8 will turn this caspase homology domain into subunit p18 and p10, which could interact with other caspase family members [22]. This domain has been reported to be the key part of caspase-8, and most *CASP8* mutations are inactivating mutations [23]. Thus, suppression of apoptosis caused by *CASP8* mutations may contribute to OSCC pathogenesis.

*CDKN2A* encodes two proteins, p16 and p14, both of which act as tumor suppressors through regulation of the cell cycle. These have been investigated in HNSCC, and most *CDKN2A* mutations are inactivating mutations [3, 24–26]. Based on the transcription of *CDKN2A*, we deduced that most of the *CDKN2A* mutations were in the ankyrin repeats of p16. The dysregulation of the cell cycle caused by *CDKN2A* mutations may also lead to OSCC development.

There are some limitations in our study. The sample size was relatively small, and therefore the mutation frequencies reported here might not be fully representative of a larger population. Thus, a larger sample size is recommended in the future studies. In addition, we only used one high-throughput next-generation sequencing platform for mutation analysis, so other sequencing platforms, validation methods (such as digital PCR assays and Custom TaqMan SNP Genotyping assays) and independent cohorts are needed to validate our findings.

## Conclusions

Our results indicate that *TP53*, *NOTCH1*, *CASP8*, *CDKN2A*, and *CDH1* were the most commonly mutated genes in a cohort of OSCC patients treated at our center. The threshold of allele frequency does not affect the results of prognostic analysis using deep NGS. Further investigation with larger sample sizes are suggested to fully determine the most appropriate allele threshold for NGS studies of OSCC.

## Additional files

Additional file 1: **Table S1.** Non-synonymous mutations (threshold of allele frequency of ≥10%) in the cancerous tissues from oral squamous cell carcinoma patients. (DOCX 22 kb)

Additional file 2: **Table S2.** Non-synonymous mutations (threshold of allele frequency of ≥5%) in the cancerous tissues from oral squamous cell carcinoma patients. (DOCX 23 kb)

Additional file 3: **Table S3.** Non-synonymous mutations (threshold of allele frequency of ≥3%) in the cancerous tissues from oral squamous cell carcinoma patients. (DOCX 24 kb)

Additional file 4: **Table S4.** Validation of TP53 mutations by Sanger sequencing in patients with oral squamous cell carcinoma. *NA: the DNA is not available. (DOCX 16 kb)

Additional file 5: **Figure S1.** Survival comparison between patients with non-synonymous mutations (including all targeted genes of CAGE) and wild type using the threshold of allele frequency of 5%, and the difference was not significant with regard to overall survival (**A**), disease-free survival (**B**), locoregional recurrence-free survival (**C**), and distant metastasis-free survival (**D**). (JPG 285 kb)

Additional file 6: **Figure S2.** Survival comparison between patients with non-synonymous mutations (including all targeted genes of CAGE) and wild type using the threshold of allele frequency of 3%, and the difference was not significant with regard to overall survival (**A**), disease-free survival (**B**), locoregional recurrence-free survival (**C**), and distant metastasis-free survival (**D**). (JPG 280 kb)

Additional file 7: **Table S5.** Correlation between TP53 non-synonymous mutation and baseline characteristics in patients with oral squamous cell carcinoma. (DOCX 19 kb)

Additional file 8: **Table S6.** Correlation between NOTCH1 non-synonymous mutation and baseline characteristics in patients with oral squamous cell carcinoma. (DOCX 19 kb)

Additional file 9: **Table S7.** Correlation between CAPS8 non-synonymous mutation and baseline characteristics in patients with oral squamous cell carcinoma. (DOCX 19 kb)

Additional file 10: **Table S8.** Correlation between CDKN2A non-synonymous mutation and baseline characteristics in patients with oral squamous cell carcinoma. (DOCX 19 kb)

Additional file 11: **Table S9.** Correlation between CDH1 non-synonymous mutation and baseline characteristics in patients with oral squamous cell carcinoma. (DOCX 19 kb)

## Abbreviations

CAGE: Cancer associated gene exons
DFS: Disease-free survival
DMFS: Distant metastasis-free survival
FFPE: Formalin-fixed and paraffin-embedded
HNSCC: Head and neck squamous cell carcinoma
LRFS: Locoregional recurrence-free survival
NGS: Next-generation sequencing
OS: Overall survival
OSCC: Oral squamous cell carcinoma
TCGA: The Cancer Genome Atlas
