# Supplementary material for: Mutation allele frequency threshold does not affect prognostic analysis using next-generation sequencing in oral squamous cell carcinoma
Source: BMC Cancer. 2018 Jul 24;18:758. doi: 10.1186/s12885-018-4481-8 (PMC6057048; doi:10.1186/s12885-018-4481-8)
Supplement: Supplementary file 1 — Table S1. Non-synonymous mutations (threshold of allele frequency of ≥10%) in the cancerous tissues from oral squamous cell carcinoma patients. (DOCX 22 kb) [file 12885_2018_4481_MOESM1_ESM.docx]

**Table S1.** Non-synonymous mutations (threshold of allele frequency of ≥10%) in the cancerous tissues from oral squamous cell carcinoma patients

| Patient | *TP53* | *NOTCH1* | *CDKN2A* | *CASP8* | *CDH1* | *EGFR* | *IGFBP3* | *ANXA1* | *TGFB1* | *TP63* | *CTNNB1* | *PTEN* |
| --- | --- | --- | --- | --- | --- | --- | --- | --- | --- | --- | --- | --- |
| 1 | p.Pro128Ser  p.Leu93fs | p.Pro2325Leu  splicesite_5  p.Glu1866Lys  p.Asp1835Asn  p.Arg1758Cys  p.Arg1211Gln  p.Asp965Asn  p.Thr588Ile  p.Ala355Thr  p.Asp338Asn | wt | wt | p.Gln422Ter | p.Ala237Val  p.Thr302Ile  splicesite_3 | p.His290Tyr  p.Gly78Ser | wt | p.Cys223Tyr | wt | wt | wt |
| 2 | p.Arg175His | p.Phe436Leu | wt | wt | wt | wt | wt | wt | wt | wt | wt | wt |
| 3 | p.Cys135Phe | p.Cys1142Tyr | wt | wt | wt | wt | wt | wt | wt | wt | wt | wt |
| 4 | p.Arg282Trp | p.Thr767Ile  p.Pro67Leu | wt | wt | wt | wt | wt | wt | wt | wt | wt | wt |
| 5 | p.Pro152Leu | p.Cys1142Tyr | wt | wt | wt | wt | wt | wt | wt | wt | wt | wt |
| 6 | p.Val272Leu | p.Glu455Val | wt | wt | wt | wt | wt | wt | wt | wt | wt | wt |
| 7 | p.Tyr220Cys  p.Ser15Ile | p.Ala831Ser | wt | wt | wt | wt | wt | wt | wt | wt | wt | wt |
| 8 | p.Arg337Cys  p.Arg282Trp | p.Glu488Lys | wt | p.Ile354Asn  p.Lys532fs | wt | wt | wt | wt | wt | wt | wt | wt |
| 9 | p.Pro151Thr | wt | wt | p.Arg472Ter | p.Thr646Ser | wt | wt | wt | wt | wt | wt | wt |
| 10 | p.Arg175His | wt | wt | p.Arg494Ter | wt | wt | wt | p.Asp197Ala | wt | wt | wt | wt |
| 11 | p.Arg282Trp | wt | wt | p.Cys404Tyr | wt | wt | wt | wt | wt | wt | wt | wt |
| 12 | p.Ile255Phe  p.Pro152Leu  p.Ser15Ile | wt | wt | wt | wt | wt | wt | wt | wt | wt | wt | wt |
| 13 | p.Glu326fs  p.Val218Glu | wt | wt | wt | wt | wt | wt | wt | wt | wt | wt | wt |
| 14 | p.Val216Met | wt | p.Glu120Ter | p.Gln108Ter | wt | wt | wt | wt | wt | wt | wt | wt |
| 15 | p.His178fs | wt | p.Glu120Ter | wt | wt | wt | wt | wt | wt | wt | wt | wt |
| 16 | p.His179Leu | p.Asp2091Tyr | wt | wt | wt | wt | wt | wt | wt | wt | wt | wt |
| 17 | p.Arg213Ter | wt | wt | wt | p.Asp854Tyr | wt | wt | wt | wt | wt | wt | wt |
| 18 | p.Phe113Cys | wt | wt | wt | wt | wt | wt | wt | wt | wt | wt | wt |
| 19 | p.Arg273His | wt | wt | wt | wt | wt | wt | wt | wt | wt | wt | wt |
| 20 | p.Arg248Gln  p.Cys176Phe | wt | wt | wt | wt | wt | wt | wt | wt | wt | wt | wt |
| 21 | p.His193Leu | wt | wt | wt | wt | wt | wt | wt | wt | wt | wt | wt |
| 22 | p.Gly245Ser | wt | wt | wt | wt | wt | wt | wt | wt | wt | wt | wt |
| 23 | p.Cys135Tyr | wt | wt | wt | wt | wt | wt | wt | wt | wt | wt | wt |
| 24 | p.Pro151His | wt | wt | wt | wt | wt | wt | wt | wt | wt | wt | wt |
| 25 | p.Arg213Gln | wt | wt | wt | wt | wt | wt | wt | wt | wt | wt | wt |
| 26 | p.Glu286Lys  p.Pro191del | wt | wt | wt | wt | wt | wt | wt | wt | wt | wt | wt |
| 27 | p.Val274Phe | wt | wt | wt | wt | wt | wt | wt | wt | wt | wt | wt |
| 28 | p.Thr253Ile  p.Asp184His  p.Cys135Phe | wt | splicesite_5 | wt | wt | wt | wt | wt | wt | wt | wt | wt |
| 29 | p.Arg342Ter | p.Ala646fs | wt | wt | wt | wt | wt | wt | wt | wt | wt | wt |
| 30 | wt | p.Asn718Ser | wt | wt | wt | wt | wt | wt | wt | wt | wt | wt |
| 31 | p.Lys319fs | p.Asn959fs  p.Asn956Lys | wt | wt | wt | wt | wt | wt | wt | wt | wt | wt |
| 32 | splicesite_3 | p.Ala465Thr | wt | wt | wt | wt | wt | wt | wt | wt | wt | wt |
| 33 | wt | p.Arg365Cys | wt | wt | wt | wt | wt | wt | wt | wt | wt | wt |
| 34 | wt | wt | wt | p.Gln417Ter  p.Gln521fs | wt | wt | wt | wt | wt | wt | wt | wt |
| 35 | p.Gln331Ter | wt | wt | wt | wt | wt | wt | wt | wt | wt | wt | wt |
| 36 | splicesite_5 | wt | p.Tyr129His | wt | wt | wt | wt | wt | wt | wt | wt | wt |
| 37 | wt | wt | p.Met52fs | wt | wt | wt | wt | wt | wt | wt | wt | wt |
| 38 | splicesite_5 | wt | wt | wt | wt | wt | wt | wt | wt | wt | wt | wt |
| 39 | splicesite_3 | wt | wt | wt | wt | wt | wt | wt | wt | wt | wt | wt |
| 40 | p.Trp53Ter | wt | wt | wt | wt | wt | wt | wt | wt | wt | wt | wt |
| 41 | wt | wt | wt | wt | wt | wt | wt | wt | wt | wt | wt | wt |
| 42 | wt | wt | wt | wt | wt | wt | wt | wt | wt | wt | wt | wt |
| 43 | wt | wt | wt | wt | wt | wt | wt | wt | wt | wt | wt | wt |
| 44 | wt | wt | wt | wt | wt | wt | wt | wt | wt | wt | wt | wt |
| 45 | wt | wt | wt | wt | wt | wt | wt | wt | wt | wt | wt | wt |
| 46 | wt | wt | wt | wt | wt | wt | wt | wt | wt | wt | wt | wt |
