# Supplementary material for: Mutation allele frequency threshold does not affect prognostic analysis using next-generation sequencing in oral squamous cell carcinoma
Source: BMC Cancer. 2018 Jul 24;18:758. doi: 10.1186/s12885-018-4481-8 (PMC6057048; doi:10.1186/s12885-018-4481-8)
Supplement: Supplementary file 3 — Table S3. Non-synonymous mutations (threshold of allele frequency of ≥3%) in the cancerous tissues from oral squamous cell carcinoma patients. (DOCX 24 kb) [file 12885_2018_4481_MOESM3_ESM.docx]

**Table S3.** Non-synonymous mutations (threshold of allele frequency of ≥3%) in the cancerous tissues from oral squamous cell carcinoma patients

| Patient | *TP53* | *NOTCH1* | *CDKN2A* | *CASP8* | *CDH1* | *EGFR* | *IGFBP3* | *ANXA1* | *TGFB1* | *TP63* | *CTNNB1* | *PTEN* |
| --- | --- | --- | --- | --- | --- | --- | --- | --- | --- | --- | --- | --- |
| 1 | p.Pro128Ser  p.Leu93fs  p.Pro85Ser | p.Pro2325Leu  splicesite_5  p.Glu1866Lys  p.Asp1835Asn  p.Arg1758Cys  p.Arg1211Gln  p.Asp965Asn  p.Thr588Ile  p.Ala355Thr  p.Asp338Asn  p.Gly1422Glu  p.Arg1356His  p.Glu569Gly  p.Thr194Ile  p.Ala2250Val  p.Asp1835Asn  p.Arg1287His | p.Pro38Ser | p.Pro152Ala  p.Glu204Lys  p.Pro27Ser  p.Gly37Glu  p.Gln274Ter  p.Ser364Asn | p.Gln422Ter  p.Pro25Ser  p.Gly81Asp  p.Arg90Trp  p.Ala359Thr  p.Pro593Leu  p.Gly239Glu  p.Gln307Lys  p.Pro404Leu  p.Val454Ile | p.Ala237Val  p.Thr302Ile  splicesite_3  p.Ser1064Asn  p.Ala118Thr  p.Gly283Ser  p.His584Gln  p.Gly874Ser  p.Leu1139Phe  p.Val1200Ile | p.His290Tyr  p.Gly78Ser  p.Pro259Ser | wt | p.Cys223Tyr  p.Pro256Ser  p.Ala89Thr | p.Pro653Leu  p.Asp666Asn  p.Gly635Asp  p.Val628Met  p.Pro532Leu  p.Leu519Phe  p.Pro161Leu  p.Thr144Met | p.Arg185Lys  p.Glu686Gly  p.Gly725Asp  p.Gln68Ter  p.Ala532Val  p.Val537Ile  p.Ala638Thr | wt |
| 2 | p.Arg175His | p.Phe436Leu  p.Cys1142Tyr | wt | wt | wt | wt | wt | wt | wt | wt | wt | wt |
| 3 | p.Cys135Phe | p.Cys1142Tyr | wt | wt | wt | wt | wt | wt | wt | wt | wt | wt |
| 4 | p.Arg282Trp  p.Asp148Asn  p.Asp57Asn  p.Ser46Phe | p.Thr767Ile  p.Pro67Leu  p.Gly2420Asp  p.Ser2221Phe  p.Ala2002Val  p.Ala1950Val  p.Gln1495Ter  p.Val1172Ala  p.Ser2435Cys  p.Pro2377Leu  p.Met2362Ile  p.Gly2259Asp  p.Ala2251Thr  p.Ala1743Thr  p.Ser1541Asn  p.Ser1354Asn  p.Gly1212Cys  p.Pro148Leu | wt | p.Gly34Asp | p.Glu336Lys  p.Trp638Ter  p.Val344Met  p.Ser356Asn | p.Arg334His  p.Arg1068Gln  p.Ala118Thr  p.Gly1022Ser  p.Ser1071Leu | p.Pro169Ser | wt | p.Gln233Arg  p.Glu142Lys | p.Pro613Leu | p.Gly740Ala | p.Arg161Lys  p.His185Tyr  p.Val249Met |
| 5 | p.Pro152Leu | p.Cys1142Tyr | wt | wt | p.Pro159Ser | wt | wt | wt | wt | wt | wt | wt |
| 6 | p.Val272Leu | p.Glu455Val | wt | wt | wt | wt | wt | wt | wt | wt | wt | wt |
| 7 | p.Tyr220Cys  p.Ser15Ile | p.Ala831Ser  p.Ala1696Thr  p.Ala1338Thr  p.Pro1337Ser | wt | wt | p.Pro159Ser | wt | wt | wt | wt | wt | wt | wt |
| 8 | p.Arg337Cys  p.Arg282Trp | p.Glu488Lys | wt | p.Ile354Asn  p.Lys532fs | wt | wt | wt | wt | wt | wt | wt | wt |
| 9 | p.Pro151Thr  p.Asn310Thr | wt | wt | p.Arg472Ter | p.Thr646Ser | wt | wt | wt | wt | wt | wt | wt |
| 10 | p.Arg175His | wt | wt | p.Arg494Ter | wt | wt | wt | p.Asp197Ala | wt | wt | wt | wt |
| 11 | p.Arg282Trp | wt | wt | p.Cys404Tyr | wt | wt | wt | wt | wt | wt | wt | wt |
| 12 | p.Ile255Phe  p.Pro152Leu  p.Ser15Ile | wt | wt | wt | wt | wt | wt | wt | wt | wt | wt | wt |
| 13 | p.Glu326fs  p.Val218Glu | wt | wt | wt | wt | wt | wt | wt | wt | wt | wt | wt |
| 14 | p.Val216Met | wt | p.Glu120Ter | p.Gln108Ter | wt | wt | wt | wt | wt | wt | wt | wt |
| 15 | p.His178fs | wt | p.Glu120Ter | wt | wt | wt | wt | wt | wt | wt | wt | wt |
| 16 | p.His179Leu | p.Asp2091Tyr | wt | wt | wt | wt | wt | wt | wt | wt | wt | wt |
| 17 | p.Arg213Ter | wt | wt | wt | p.Asp854Tyr | wt | wt | wt | wt | wt | wt | wt |
| 18 | p.Phe113Cys | wt | wt | wt | wt | wt | wt | wt | wt | wt | wt | wt |
| 19 | p.Arg273His | p.Arg2272Cys  p.Leu372Phe | wt | wt | wt | wt | wt | wt | wt | p.Met512Ile | wt | wt |
| 20 | p.Arg248Gln  p.Cys176Phe | wt | wt | wt | wt | wt | wt | wt | wt | wt | wt | wt |
| 21 | p.His193Leu | wt | wt | wt | wt | wt | wt | wt | wt | wt | wt | wt |
| 22 | p.Gly245Ser | wt | wt | wt | wt | wt | wt | wt | wt | wt | wt | wt |
| 23 | p.Cys135Tyr | p.Ile640Asn | wt | wt | wt | wt | wt | wt | wt | wt | wt | wt |
| 24 | p.Pro151His | wt | wt | wt | wt | wt | wt | wt | wt | wt | wt | wt |
| 25 | p.Arg213Gln | wt | wt | wt | wt | wt | wt | wt | wt | wt | wt | wt |
| 26 | p.Glu286Lys  p.Pro191del | wt | wt | wt | wt | wt | wt | wt | wt | wt | wt | wt |
| 27 | p.Val274Phe | wt | wt | wt | wt | wt | wt | wt | wt | wt | wt | wt |
| 28 | p.Thr253Ile  p.Asp184His  p.Cys135Phe | wt | splicesite_5 | wt | wt | wt | wt | wt | wt | wt | wt | wt |
| 29 | p.Arg342Ter | p.Ala646fs | wt | wt | wt | wt | wt | wt | wt | wt | wt | wt |
| 30 | wt | p.Asn718Ser | wt | wt | wt | wt | wt | wt | wt | wt | wt | wt |
| 31 | p.Lys319fs | p.Asn959fs  p.Asn956Lys | wt | wt | wt | wt | wt | wt | wt | p.Ala513Thr | wt | wt |
| 32 | splicesite_3  p.Ser376Phe  p.Ala159Val | p.Ala465Thr  p.Pro2332Leu  p.Gly583Ser  p.Ser579Phe | wt | p.Gly321Glu  p.Gly427Ser | wt | wt | p.Gly285Glu | wt | p.Pro354Ser | wt | p.Ala20Thr  p.Ala171Thr  p.Arg587Ter | wt |
| 33 | wt | p.Arg365Cys  p.Ala2331Val  p.Arg1234Gln  p.Arg2272His  p.Pro2270Gln  p.Thr1844Ile  p.Gly1376Asp  p.Val1229Ile  p.Gly1195Arg  p.His1190Tyr  p.Pro1097Ser  p.Gly21Asp | wt | wt | wt | wt | wt | wt | wt | wt | wt | wt |
| 34 | wt | p.Cys1142Tyr | wt | p.Gln417Ter  p.Gln521fs | wt | wt | wt | wt | wt | wt | wt | wt |
| 35 | p.Gln331Ter | p.Ser1181Phe  p.Ser2336Asn  p.Pro2325Leu  p.Gly2287Asp  p.Ser1919Asn  p.Ser1889Phe  p.Val1876Ile  p.Leu1712Phe  p.Ala1552Val  p.Pro1337Ser  p.Glu1102Lys  p.Ser836Asn  p.Pro668Ser  p.His562Tyr  p.Gly92Asp | p.Val106Met | wt | wt | wt | wt | wt | p.Asp235Asn | wt | wt | wt |
| 36 | splicesite_5 | wt | p.Tyr129His | wt | wt | wt | wt | wt | wt | wt | wt | wt |
| 37 | wt | wt | p.Met52fs | wt | wt | wt | wt | wt | wt | wt | wt | wt |
| 38 | splicesite_5 | wt | wt | wt | wt | wt | wt | wt | wt | wt | wt | wt |
| 39 | splicesite_3 | p.Gly2195Asp  p.Pro1191Leu | wt | wt | wt | wt | wt | wt | wt | wt | wt | wt |
| 40 | p.Trp53Ter | wt | wt | wt | wt | wt | wt | wt | wt | wt | wt | wt |
| 41 | wt | wt | wt | wt | wt | wt | wt | wt | wt | wt | wt | wt |
| 42 | wt | wt | wt | wt | wt | wt | wt | wt | wt | wt | wt | wt |
| 43 | wt | p.Cys1142Tyr | wt | wt | wt | wt | wt | wt | wt | wt | wt | wt |
| 44 | wt | wt | wt | wt | wt | wt | wt | wt | wt | wt | wt | wt |
| 45 | wt | wt | wt | wt | wt | wt | wt | wt | wt | wt | wt | wt |
| 46 | wt | p.Arg1044Lys | wt | wt | wt | wt | wt | wt | wt | wt | wt | wt |
