# Supplementary material for: Mutation allele frequency threshold does not affect prognostic analysis using next-generation sequencing in oral squamous cell carcinoma
Source: BMC Cancer. 2018 Jul 24;18:758. doi: 10.1186/s12885-018-4481-8 (PMC6057048; doi:10.1186/s12885-018-4481-8)
Supplement: Supplementary file 4 — Table S4. Validation of TP53 mutations by Sanger sequencing in patients with oral squamous cell carcinoma. *NA: the DNA is not available. (DOCX 16 kb) [file 12885_2018_4481_MOESM4_ESM.docx]

**Table S4.** Validation of *TP53* mutations by Sanger sequencing in patients with oral squamous cell carcinoma

| Patient | *TP53* mutation | Validation (Yes/No/NA*) | |  |
| --- | --- | --- | --- | --- |
| 1 | p.Pro128Ser  p.Leu93fs | | No  NA | |
| 2 | p.Arg175His | | Yes | |
| 3 | p.Cys135Phe | | Yes | |
| 4 | p.Arg282Trp | | Yes | |
| 5 | p.Pro152Leu | | Yes | |
| 6 | p.Val272Leu | | No | |
| 7 | p.Tyr220Cys  p.Ser15Ile | | Yes  No | |
| 8 | p.Arg337Cys  p.Arg282Trp | | No  No | |
| 9 | p.Pro151Thr | | Yes | |
| 10 | p.Arg175His | | NA | |
| 11 | p.Arg282Trp | | Yes | |
| 12 | p.Ile255Phe  p.Pro152Leu  p.Ser15Ile | | No  Yes  No | |
| 13 | p.Glu326fs  p.Val218Glu | | NA  Yes | |
| 14 | p.Val216Met | | Yes | |
| 15 | p.His178fs | | NA | |
| 16 | p.His179Leu | | No | |
| 17 | p.Arg213Ter | | Yes | |
| 18 | p.Phe113Cys | | NA | |
| 19 | p.Arg273His | | NA | |
| 20 | p.Arg248Gln  p.Cys176Phe | | Yes  Yes | |
| 21 | p.His193Leu | | Yes | |
| 22 | p.Gly245Ser | | NA | |
| 23 | p.Cys135Tyr | | Yes | |
| 24 | p.Pro151His | | No | |
| 25 | p.Arg213Gln | | Yes | |
| 26 | p.Glu286Lys  p.Pro191del | | Yes  NA | |
| 27 | p.Val274Phe | | Yes | |
| 28 | p.Thr253Ile  p.Asp184His  p.Cys135Phe | | Yes  Yes  Yes | |
| 29 | p.Arg342Ter | | Yes | |
| 30 | wt | |  | |
| 31 | p.Lys319fs | | NA | |
| 32 | splicesite_3 | | No | |
| 33 | wt | |  | |
| 34 | wt | |  | |
| 35 | p.Gln331Ter | | Yes | |
| 36 | splicesite_5 | | Yes | |
| 37 | wt | |  | |
| 38 | splicesite_5 | | Yes | |
| 39 | splicesite_3 | | No | |
| 40 | p.Trp53Ter | | Yes | |
| 41 | wt | |  | |
| 42 | wt | |  | |
| 43 | wt | |  | |
| 44 | wt | |  | |
| 45 | wt | |  | |
| 46 | wt | |  | |

*NA: the DNA is not available.
