# Supplementary figures and images for: Mutation allele frequency threshold does not affect prognostic analysis using next-generation sequencing in oral squamous cell carcinoma
Source: BMC Cancer. 2018 Jul 24;18:758. doi: 10.1186/s12885-018-4481-8 (PMC6057048; doi:10.1186/s12885-018-4481-8)

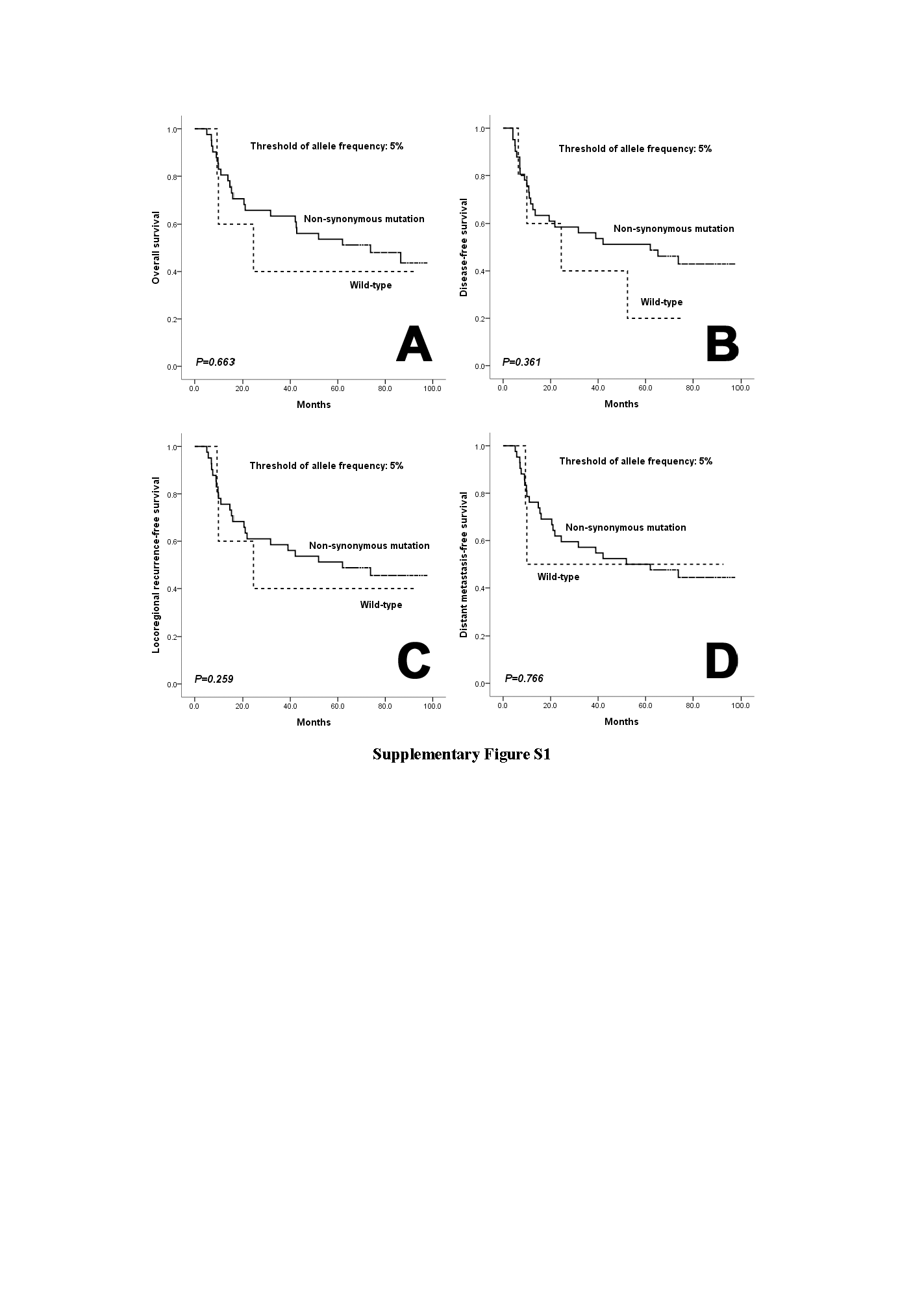

Supplement: Supplementary file 5 — Figure S1. Survival comparison between patients with non-synonymous mutations (including all targeted genes of CAGE) and wild type using the threshold of allele frequency of 5%, and the difference was not significant with regard to overall survival (A), disease-free survival (B), locoregional recurrence-free survival (C), and distant metastasis-free survival (D). (JPG 285 kb) [file 12885_2018_4481_MOESM5_ESM.jpg]

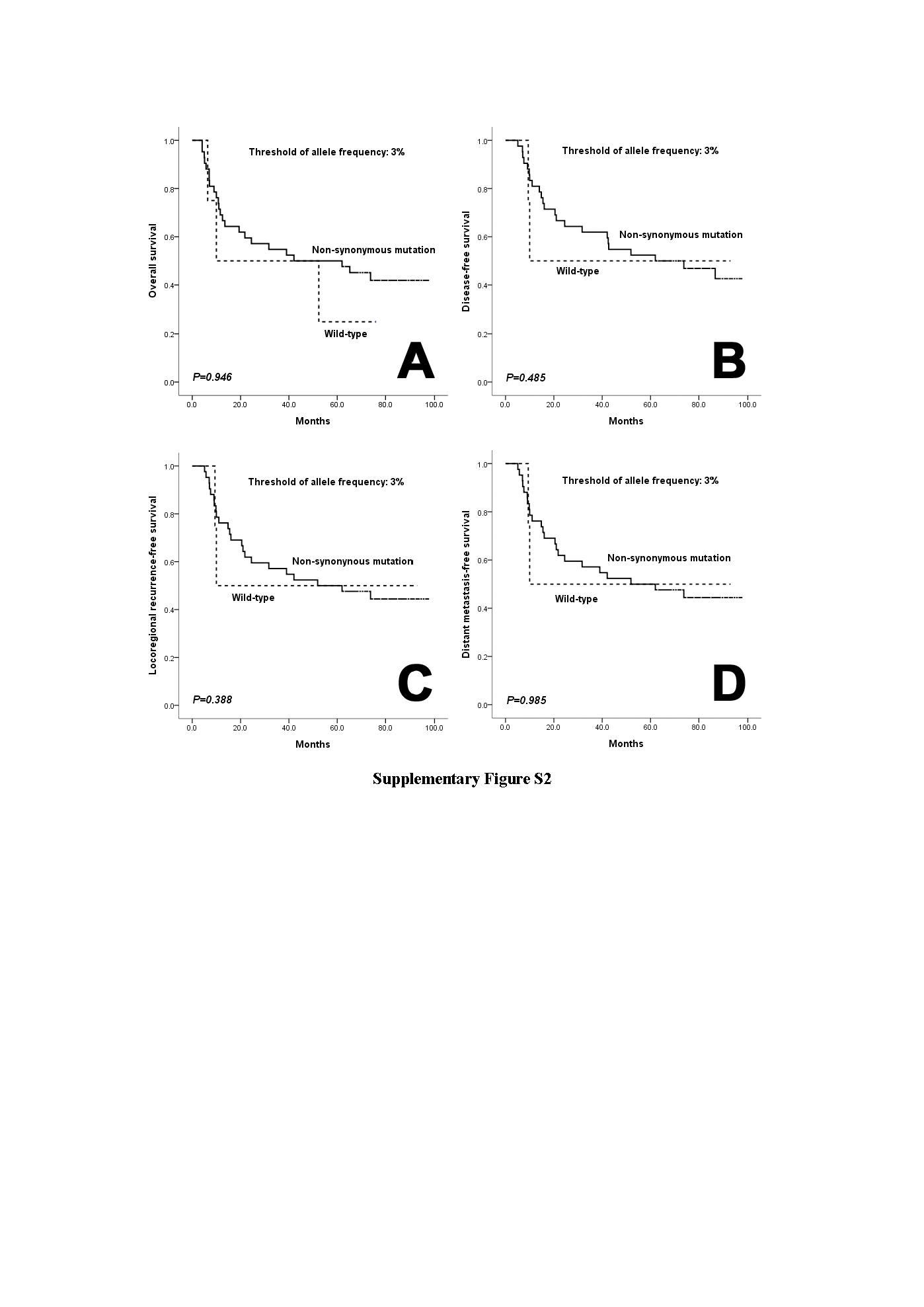

Supplement: Supplementary file 6 — Figure S2. Survival comparison between patients with non-synonymous mutations (including all targeted genes of CAGE) and wild type using the threshold of allele frequency of 3%, and the difference was not significant with regard to overall survival (A), disease-free survival (B), locoregional recurrence-free survival (C), and distant metastasis-free survival (D). (JPG 280 kb) [file 12885_2018_4481_MOESM6_ESM.jpg]
