# Supplementary material for: Mutation allele frequency threshold does not affect prognostic analysis using next-generation sequencing in oral squamous cell carcinoma
Source: BMC Cancer. 2018 Jul 24;18:758. doi: 10.1186/s12885-018-4481-8 (PMC6057048; doi:10.1186/s12885-018-4481-8)
Supplement: Supplementary file 7 — Table S5. Correlation between TP53 non-synonymous mutation and baseline characteristics in patients with oral squamous cell carcinoma. (DOCX 19 kb) [file 12885_2018_4481_MOESM7_ESM.docx]

**Table S5.** Correlation between *TP53* non-synonymous mutation and baseline characteristics in patients with oral squamous cell carcinoma

| Characteristics | Total patients N=46 | Non-synonymous mutations | | P value* |
| --- | --- | --- | --- | --- |
|  |  | + | - |  |
|  | n (%) | n (%) | n (%) |  |
| Gender | | | | |
| Male | 12 (26.1) | 10 (27.8) | 2 (20.0) | 1.000 |
| Female | 34 (73.9) | 26 (72.2) | 8 (80.0) |  |
| Age (years) | | | | |
| <60 | 27 (58.7) | 22 (61.1) | 5 (50.0) | 0.719 |
| ≥60 | 19 (41.3) | 14 (38.9) | 5 (50.0) |  |
| Site | | | | |
| Tongue | 19 (41.3) | 16 (44.4) | 3 (30.0) | 0.791 |
| Buccal | 6 (13.0) | 5 (13.9) | 1 (10.0) |  |
| Gingiva | 6 (13.0) | 5 (13.9) | 1 (10.0) |  |
| Floor of mouth | 3 (6.5) | 2 (5.6) | 1 (10.0) |  |
| Palate | 9 (19.6) | 6 (16.7) | 3 (30.0) |  |
| Retromolar trigone | 3 (6.5) | 2 (5.6) | 1 (10.0) |  |
| Clinical T stage | | | | |
| T1/T2 | 13 (28.3) | 9 (25.0) | 4 (40.0) | 0.435 |
| T3/T4 | 33 (71.7) | 27 (75.0) | 6 (60.0) |  |
| Clinical N stage | | | | |
| N0 | 14 (30.4) | 10 (27.8) | 4 (40.0) | 0.436 |
| N1 | 12 (26.1) | 11 (30.6) | 1 (10.0) |  |
| N2 | 20 (43.5) | 15 (41.7) | 5 (50.0) |  |
| Clinical stage |  |  |  |  |
| III | 22 (47.8) | 19 (52.8) | 3 (30.0) | 0.289 |
| IVA | 24 (52.2) | 17 (47.2) | 7 (70.0) |  |
| Pathological differentiation grade | | | | |
| Well | 13 (28.3) | 10 (27.8) | 3 (30.0) | 1.000 |
| Moderately/Poorly | 33 (71.7) | 26 (72.2) | 7 (70.0) |  |
| Smoking status** | | | | |
| Current/former | 19 (41.3) | 16 (44.4) | 3 (30.0) | 0.488 |
| Never | 27 (58.7) | 20 (55.6) | 7 (70.0) |  |
| Alcohol use*** | | | | |
| Positive | 24 (52.2) | 21 (58.3) | 3 (30.0) | 0.159 |
| Negative | 22 (47.8) | 15 (41.7) | 7 (70.0) |  |
| * *P* value from the chi-square test was reported to compare the difference between the patients with and without *TP53* non-synonymous mutation based on different baseline characteristics.  **Former/current smokers defined as at least a one pack-year history of smoking.  ***Positive alcohol use was defined as current alcohol use of more than one drink per day for 1 year (12 ounces of beer with 5% alcohol, or 5 ounces of wine with 12%-15% alcohol, or one ounce of liquor with 45%-60% alcohol). All other patients were classified as negative alcohol use. | | | | |
