# Supplementary material for: Mutation allele frequency threshold does not affect prognostic analysis using next-generation sequencing in oral squamous cell carcinoma
Source: BMC Cancer. 2018 Jul 24;18:758. doi: 10.1186/s12885-018-4481-8 (PMC6057048; doi:10.1186/s12885-018-4481-8)
Supplement: Supplementary file 8 — Table S6. Correlation between NOTCH1 non-synonymous mutation and baseline characteristics in patients with oral squamous cell carcinoma. (DOCX 19 kb) [file 12885_2018_4481_MOESM8_ESM.docx]

**Table S6.** Correlation between *NOTCH1* non-synonymous mutation and baseline characteristics in patients with oral squamous cell carcinoma

| Characteristics | Total patients N=46 | Non-synonymous mutations | | P value* |
| --- | --- | --- | --- | --- |
|  |  | + | - |  |
|  | n (%) | n (%) | n (%) |  |
| Gender | | | | |
| Male | 12 (26.1) | 3 (21.4) | 9 (28.1) | 0.729 |
| Female | 34 (73.9) | 11 (78.6) | 23 (71.9) |  |
| Age (years) | | | | |
| <60 | 27 (58.7) | 7 (50.0) | 20 (62.5) | 0.428 |
| ≥60 | 19 (41.3) | 7 (50.0) | 12 (37.5) |  |
| Site | | | | |
| Tongue | 19 (41.3) | 6 (40.0) | 13 (41.9) | 0.028 |
| Buccal | 6 (13.0) | 3 (20.0) | 3 (9.7) |  |
| Gingiva | 6 (13.0) | 0 (0) | 6 (19.4) |  |
| Floor of mouth | 3 (6.5) | 1 (6.7) | 2 (6.5) |  |
| Palate | 9 (19.6) | 1 (6.7) | 8 (25.0) |  |
| Retromolar trigone | 3 (6.5) | 3 (20.0) | 0 (0) |  |
| Clinical T stage | | | | |
| T1/T2 | 13 (28.3) | 7 (50.0) | 6 (18.8) | 0.041 |
| T3/T4 | 33 (71.7) | 7 (50.0) | 26 (81.2) |  |
| Clinical N stage | | | | |
| N0 | 14 (30.4) | 2 (14.3) | 12 (37.5) | 0.301 |
| N1 | 12 (26.1) | 4 (28.6) | 8 (25.0) |  |
| N2 | 20 (43.5) | 8 (57.1) | 12 (37.5) |  |
| Clinical stage |  |  |  |  |
| III | 22 (47.8) | 5 (35.7) | 17 (53.1) | 0.277 |
| IVA | 24 (52.2) | 9 (64.3) | 15 (46.9) |  |
| Pathological differentiation grade | | | | |
| Well | 13 (28.3) | 2 (14.3) | 11 (34.4) | 0.286 |
| Moderately/Poorly | 33 (71.7) | 12 (85.7) | 21 (65.6) |  |
| Smoking status** | | | | |
| Current/former | 19 (41.3) | 5 (35.7) | 14 (43.8) | 0.611 |
| Never | 27 (58.7) | 9 (64.3) | 18 (56.3) |  |
| Alcohol use*** | | | | |
| Positive | 24 (52.2) | 8 (57.1) | 16 (50.0) | 0.655 |
| Negative | 22 (47.8) | 6 (42.9) | 16 (50.0) |  |
| * *P* value from the chi-square test was reported to compare the difference between the patients with and without *NOTCH1* non-synonymous mutation based on different baseline characteristics.  **Former/current smokers defined as at least a one pack-year history of smoking.  ***Positive alcohol use was defined as current alcohol use of more than one drink per day for 1 year (12 ounces of beer with 5% alcohol, or 5 ounces of wine with 12%-15% alcohol, or one ounce of liquor with 45%-60% alcohol). All other patients were classified as negative alcohol use. | | | | |
